# Machine learning-based detection of electrosurgical device-induced bleeding in laparoscopic videos of diaphragmatic hernia repair

**DOI:** 10.1007/s00464-025-12078-5

**Published:** 2025-08-18

**Authors:** Vincent J. Ribbens, Simon C. Baltus, Can Ozan Tan, Ivo A. M. J. Broeders

**Affiliations:** 1 Surgery Department, Meander Medical Centre, Maatweg, 3818 TZ Amersfoort, Utrecht The Netherlands; 2 Robotics and Mechatronics, University of Twente, Drienerlolaan, 5722 NB Enschede, Overijssel The Netherlands

**Keywords:** Electrosurgery, Bleedings, Skills assessment, Machine learning

## Abstract

**Background:**

Electrosurgical devices provide significant advantages for tissue dissection in laparoscopic procedures. However, achieving optimal hemostasis while minimizing tissue coagulation is challenging. Monitoring device-induced bleeding will provide viable information for surgical skills assessment. We aimed to automatically detect bleeding induced by electrosurgical device use in laparoscopic videos using machine learning.

**Methods:**

We present a two-step methodology for the automated detection of device-induced bleeding. First, based on the color representation, a random forest classifier (RFC) detects blood pixels in the frames before and after the electrosurgical device activation. Subsequently, a logistic regression (LR) model decides whether bleeding has occurred based on the change in blood pixels. The moments of device activations during surgery can be extracted automatically by a synchronized recording of the laparoscopic video and energy generator data. The RFC and LR were developed on the manual annotation of 34 images and 2678 video fragments from forty-five patients who underwent diaphragmatic hernia repair between May 2023 and October 2024. The performance of the RFC was evaluated by an 80/20 split for training and testing, while a stratified threefold cross-validation assessed the LR performance.

**Results:**

The blood pixel detection showed an accuracy of 94% and a Dice score of 0.472. The classification of automatically extracted video fragments showed that device-induced bleeding can be detected with a 78.2% accuracy, 4.6% precision, 78.1% specificity, and 81.0% sensitivity.

**Conclusion:**

The presented work on device-induced bleeding detection is a step toward quantifying the effect of electrosurgery use. We showed a machine learning-based methodology that accurately identifies video fragments of device activations without bleeding but struggles to identify bleeding precisely. Future work should focus on developing device-induced bleeding detection in a larger, more diverse dataset.

Electrosurgical devices are standard surgical instruments in laparoscopic procedures, providing significant benefits for tissue dissection. However, achieving hemostasis while minimizing tissue coagulation can be challenging. In addition, inadequate device application can lead to adverse events, such as intraoperative bleeding or damage to surrounding tissue [1, 2].

An energy dashboard, providing post-operative insights into electrosurgical device usage, has recently been introduced as a new methodology for surgical skills assessment [3]. The digital tool quantifies activation frequency, duration, and total applied energy by integrating the intra-operative video with energy generator data. Monitoring device-induced bleeding would complement this assessment as it provides crucial information for improving the device application and targeted training [4].

Analysis of technical surgical skills in laparoscopic videos through artificial intelligence is a growing field in surgical education [5]. Currently, the assessment of laparoscopic videos mainly focuses on the movement patterns of tools. Recent studies also assessed the automated detection of blood in laparoscopic videos [6, 7]. However, limited studies evaluated the detection of bleeding onset related to tool usage [8].

We aimed to improve surgical skills assessment by automatically detecting bleeding induced by electrosurgical device activation. To this end, we developed a machine learning algorithm for the automated detection of blood pixels in laparoscopic videos. Subsequently, we assessed the ability of machine learning to detect bleeding induced by tool activations.

## Methods

### Study outline

**Fig. 1:**
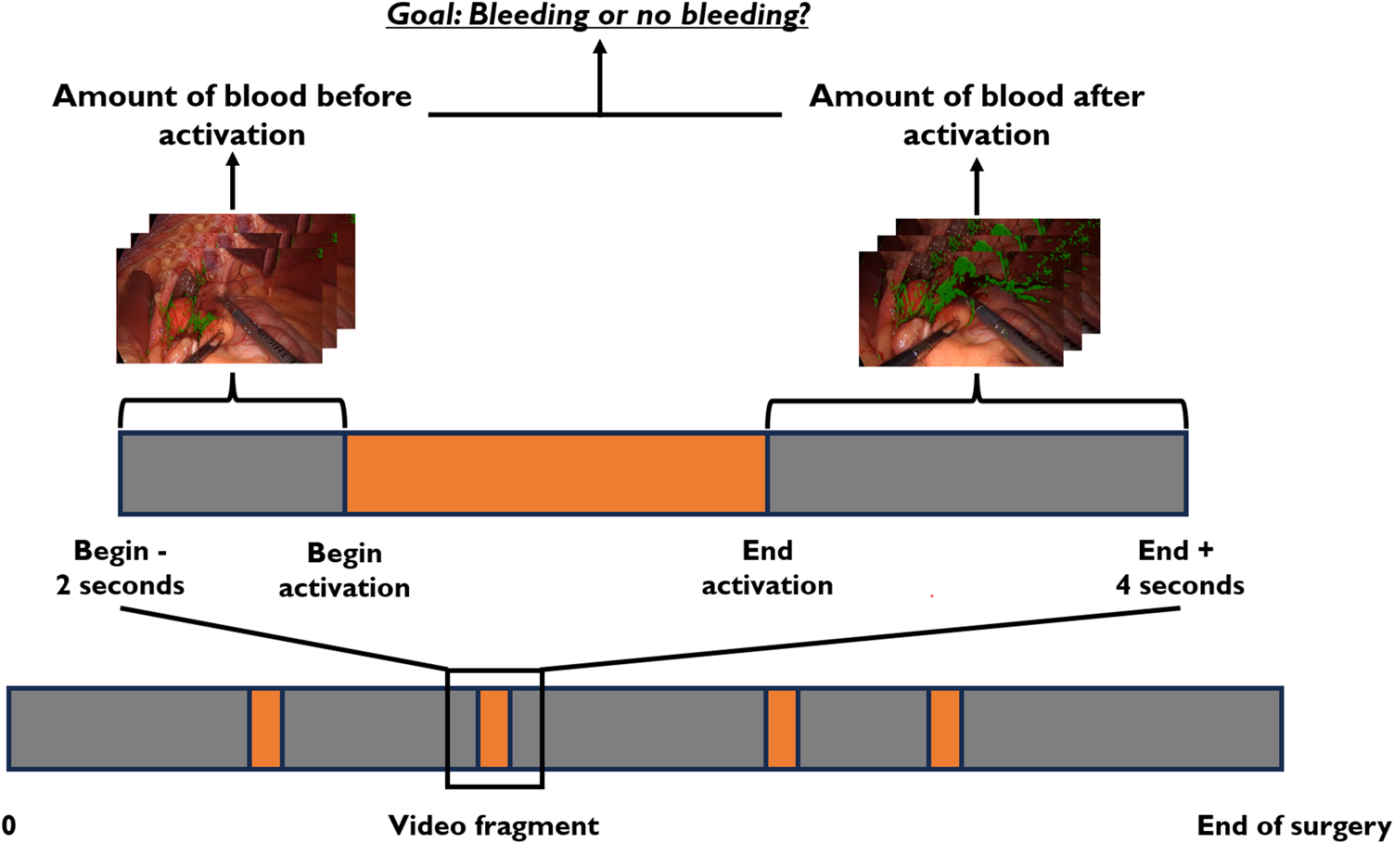
A visualization of the automated detection of device-induced bleeding. This detection is performed based on a video fragment of frames before and after activation of the electrosurgical device (activation is shown in orange). First, an RFC detects blood pixels in the laparoscopic video frames. Subsequently, bleeding can be detected by a LR classifier based on the change in the amount of detected blood within the video fragment

This study on the automated detection of device-induced bleeding involved two steps (Fig. 1). First, we developed a machine learning algorithm for the automated detection of blood pixels in laparoscopic videos. Second, a machine learning classifier was applied to the change between the blood pixels in the frames before and after activation to detect whether bleeding had occurred.

This study was performed based on data from forty-five patients who underwent diaphragmatic hernia repair between May 2023 and October 2024 at a single institution (Meander Medical Center, Amersfoort, the Netherlands). The surgeries were performed with a single electrosurgical device (ENSEAL X1 curved jaw tissue sealer, Johnson & Johnson MedTech). During surgery, simultaneous energy and laparoscopic video were recorded for each patient. The laparoscopic videos were recorded using systems from Karl Storz (Karl Storz SE & Co. KG, Tuttlingen, Germany) and Stryker (Stryker Corporation, Kalamazoo, MI, USA), with a resolution of 1920 × 1080 pixels at 50 or 60 frames per second. The energy recording was performed with a current clamp on the power cable supplying the energy generator. The time points of device activations could be extracted automatically from the energy recording. Therefore, synchronization between the energy recording and the laparoscopic video led to the automated extraction of the relevant video fragments containing the electrosurgical tool activations. The blood pixel detector used selected frames from the laparoscopic videos, whereas the device-induced bleeding detector used the extracted video fragments.

This study was conducted in accordance with the principles of the Declaration of Helsinki. All laparoscopic video and energy data were anonymized prior to analysis. This manuscript adheres to the STROBE (Strengthening the Reporting of Observational Studies in Epidemiology) guidelines to ensure transparency and completeness in the reporting.

### Automated detection of blood pixels

The blood detection algorithm used color differences to detect blood pixels in laparoscopic images [6]. First, multiple color representations served as features to distinguish blood and non-blood pixels. Subsequently, a random forest classifier (RFC) used selected color representations as features to perform the blood detection.

Blood pixels were manually annotated in thirty-four laparoscopic images from twenty-five different procedures to assess the color differences between blood and non-blood pixels. These images were purposefully selected to reflect a wide range of surgical scenes, with variations in lighting conditions, camera angles, and tissue appearance. This sampling strategy aimed to capture variability in anatomy and context. The number of images was chosen to balance the labor-intensive nature of manual blood pixel annotation with sufficient representation of relevant blood features. The annotations were performed by two physicians trained by a gastro-intestinal surgeon. Consensus between the two annotators was used to ensure blood pixels were annotated consistently. The color differences were quantified based on RGB (Red, Green, Blue), HSV (Hue, Saturation, Value), and HSL (Hue, Saturation, Light) representation systems to calculate fifteen features based on previous literature and the color wheel [6, 9]. Features were excluded based on the variance inflation factor (VIF, score below ten) and hierarchical clustering to minimize multicollinearity. The hierarchical clustering was created by Spearman’s rank-order correlation, followed by Ward’s minimum variance method. For each feature, the univariate correlation with blood was calculated using the point-biserial correlation coefficient.

Subsequently, an RFC was developed to detect blood pixels based on the selected color features. The model was trained based on twenty-eight images and tested using six images. A Bayesian hyperparameter sweep was executed to optimize the Dice score based on the number of trees, node split criterion between Gini and Entropy coefficients, maximum tree depth, and the maximum samples per tree. A threshold of 0.5 was applied to the output probabilities of the RFC to label individual pixels as blood or non-blood. Through the comparison with the manual annotations as ground truth, the performance of the best-performing model was quantified using the accuracy (overall proportion of correctly classified pixels), precision (proportion of predicted blood pixels that were correctly classified), sensitivity (proportion of true blood pixels correctly identified), specificity (proportion of non-blood pixels correctly classified), Dice score (harmonic mean of precision and sensitivity), and area (mean percentage of predicted blood pixels relative to the annotated blood area). The area metric was included to assess whether the model tended to over-segment or under-segment blood regions compared to the ground truth.

### Automated detection of a device-induced bleeding

The automated detection of device-induced bleeding focused on detecting bleeding onset after tool activation. As shown in Fig. 1, the methodology detected bleeding by comparing the change in the amount of blood in the laparoscopic video frames after to before activation. Therefore, this detection was performed based on video fragments extracted from the laparoscopic videos. Based on empirical findings, the video fragments involved frames from two seconds before tool activation up to four seconds after activation. These video fragments were automatically created using the synchronized recording of video and energy generator data. For this study, the forty-five laparoscopic videos were converted to 2678 video fragments of individual tool activations.

**Fig. 2:**
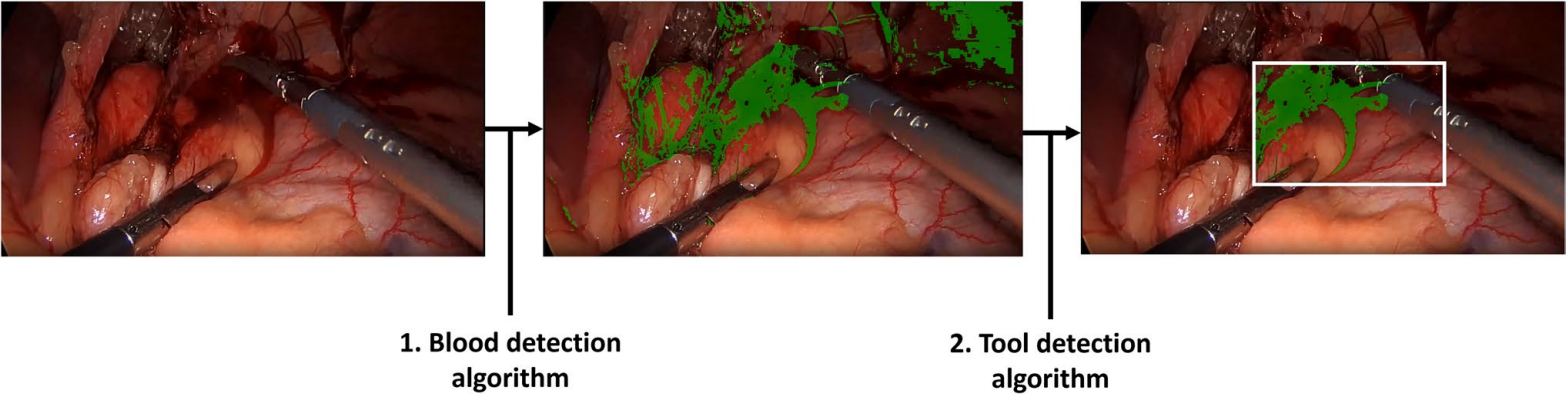
Combining pixel detection and electrosurgical tool localization leads to a localized quantification of blood in the laparoscopic video

In each video fragment, the blood in the frames before and after activation was detected by the method described in 2.2. However, the blood was only quantified around the tool location to enable a focused detection of bleeding onset. The localized detection of blood pixels was enabled by an automated electrosurgical tool detection model, which formed a region of interest (ROI) around the tool tip (Fig. 2). The ROI was frozen in the frames before and after tool activation based on the tool tip location during activation. The tool tip location was detected by a dedicated YOLO11m algorithm [10].

The YOLO11m algorithm was developed on 5730 laparoscopic images with manually annotated bounding box labels for the Enseal, graspers, irrigator, and scissors. The frames were extracted at 5-s intervals from twelve diaphragmatic hernia procedures to create tool diversity and anatomic variability. The model was trained using various configurations, optimizing for both precision and recall at intersection-over-union (IoU) thresholds of 0.5 and 0.9. A hyperparameter sweep was conducted over model architecture (nano, medium, large, xlarge), training objectives (mAP@0.5, mAP@0.5:0.95, and a combined fitness function (weighted sum of mAP@0.5, mAP@0.9, and mAP@0.5:0.95)), class weighting schemes, and the number of frozen layer. The mAP stands for mean average precision (mAP) calculated for an IoU, e.g., mAP@0.5:0.95 averages the mAP over multiple IoU thresholds with increments of 0.05.

The blood pixels detected in the ROI in the frames before and after activation were compared to assess whether the activation led to bleeding. The changes in blood presence were quantified as follows:

$$Rati{o}_{mean}=\frac{\text{Mean percentage of bl}\text{ood pixels in ROI of frames after activation}}{\text{Mean percentage of blood pixels in ROI of frames before activation}},$$

$$Rati{o}_{max}=\frac{\text{Maximum percentage of blood pixels in ROI of frames after activation}}{\text{Maximum percentage of blood pixels in ROI of frames before a}{\text{ctivation}}},$$

$$Bloo{d}_{pos{t}_{mean}}=\frac{\text{Mean number of blood pixels in ROI of frames after activation}}{\text{Total number of pixels in ROI of frames after activation}},$$

$$Bloo{d}_{mean}=\frac{\text{Mean number of blood pixels in ROI of frames after activation}}{\text{Total number of pixels in ROI of frames after activation}}-\frac{\text{Mean number of blood pixels in ROI of frames before activation}}{\text{Total number of pixels in ROI of frames before activation}},$$

$$Increas{e}_{mean}=\frac{\text{Mean blood pixels increase in ROI over frames after ac}{\text{tivation}}}{\text{Mean blood pixels increase in ROI over frames before activation}},$$

$$Increas{e}_{max}=\frac{\text{Maximum blood pixels increase in ROI over frames after activation}}{\text{Maximum blood pixels increase in ROI over frames before activation}}.$$

**Fig. 3:**
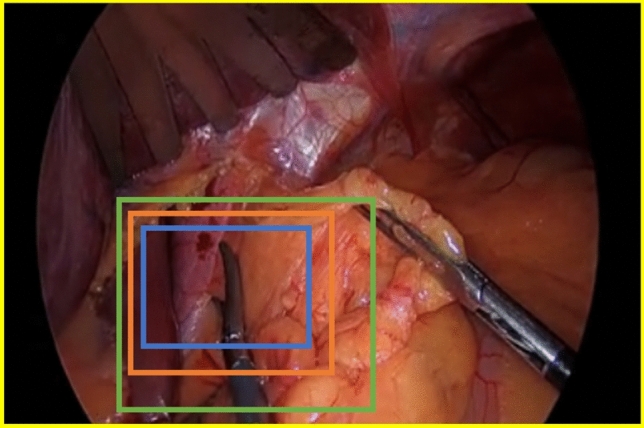
Visualization of the four ROI sizes used to evaluate the model performance. (1) Original ROI by YOLO algorithm (blue), (2) medium enlargement of 1.6 × the original ROI (orange), (3) large enlargement of 2.7 × the original ROI (green), and (4) full frame size (yellow) (Color figure online)

The six definitions of blood change were used as possible features for classification by a logistic regression (LR) model. Based on forward selection optimized for the receiver operating characteristic-area under the curve (ROC-AUC), the best features for bleeding detection were selected. The LR model was developed based on annotated video fragments, either classified as device-induced bleeding or not. Device-induced bleeding was defined as newly occurring bleeding after electrosurgical activation that required cauterization or a comparable surgical action to restore hemostasis. The classification was performed by two trained physicians who had to obtain consensus within their classifications. To assess the model performance, a stratified threefold cross-validation with class weighting was performed. For each fold, two-thirds of the 2678 annotated video fragments were used to train the model, while the remaining one-third was reserved for testing. The testing based on three data splits enabled the assessment of all patients. The stratification ensured consistent data distribution between bleeding and non-bleeding video fragments. The LR model classified video fragments as bleeding if the predicted probability exceeded a threshold of 0.5. The model’s performance was evaluated through the ROC AUC, sensitivity, specificity, accuracy, and precision. The device-induced bleeding detection performance was measured for four ROI sizes around the tool (Fig. 3) to evaluate its influence: original ROI by YOLO algorithm (Blue), medium enlargement (1.6 × ROI by YOLO, orange), large enlargement(2.7 × ROI by YOLO, green), and full frame size (1920 × 1080 pixels, yellow). When the enlarged ROI extended beyond the frame boundaries, it was clipped to remain within the frame dimensions.

## Results

### Automated detection of blood pixels

**Fig. 4:**
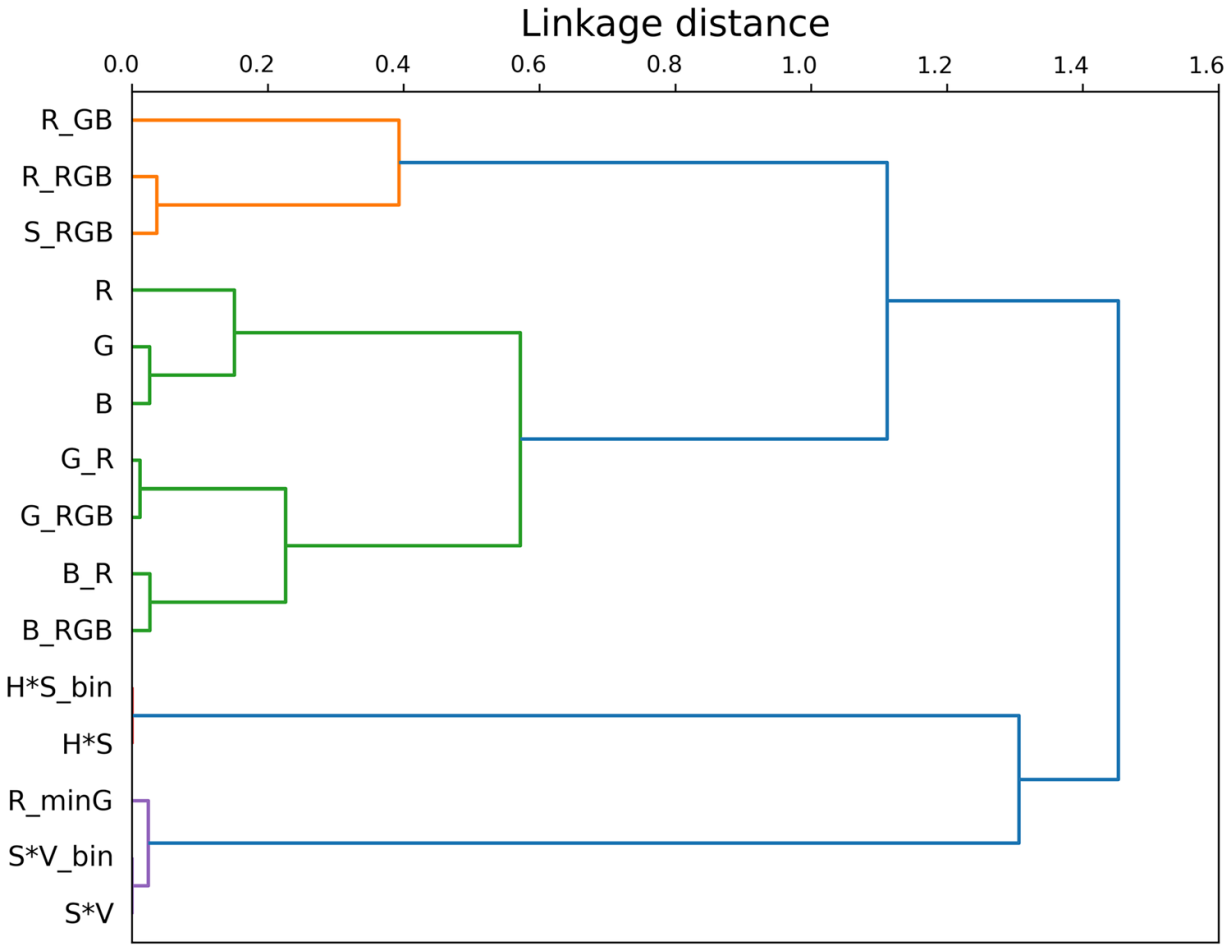
Dendrogram of all fifteen features evaluated for Spearman’s rank-order correlation. A low linkage distance indicates high correlation between features. Abbreviations: R, G, B = red, green, and blue channels; R_GB, G_RB, B_RG = ratios between individual RGB channels; R_RGB, G_RGB, B_RGB = normalized channel intensities (e.g., red divided by the sum of R + G + B); HS, SV = product of hue and saturation and saturation and value; HS_bin, SV_bin = binarized versions of the HS and SV features; R_minG = red minus green intensity (Color figure online)

By annotating thirty-four laparoscopic images, the color representations of approximately seventy million pixels were used to develop blood pixel detection by the RFC. The dendrogram in Fig. 4 provides insight into the relative multicollinearity of the color features. The correlations of the seven selected features, which all had p-values below 0.001, are given in Table 1.

**Table 1:** Point biserial correlation of the selected color features

Features	$\frac{R}{\sqrt{R+G+B}}$	$\frac{G}{\sqrt{R+G+B}}$	$\frac{B}{\sqrt{R+G+B}}$	$\frac{R}{\sqrt{G+B}}$	$R-G$	$R$	$H*S \, \text{binarized}$
Correlation	0.330	− 0.314	− 0.257	0.188	− 0.011	− 0.139	0.071

The letters in the features correspond to the channel values from the RGB and HLS color representations. “H*S binarized” refers to the binarization of the hue saturation channel product in the HSV color space

**Fig. 5:**
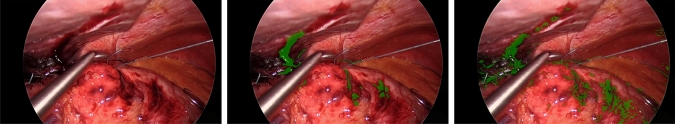
Test laparoscopic video frame with the lowest Dice score of 0.392. Left: original video frame, Middle: green overlay of the manually annotated blood (ground truth), Right: green overlay of the RFC output (prediction) (Color figure online)

**Fig. 6:**
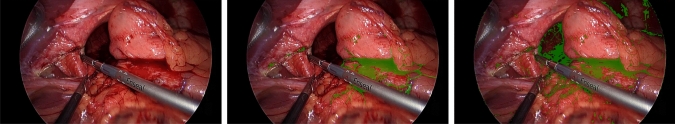
Test laparoscopic video frame with the highest Dice score of 0.541. Left: original video frame, Middle: green overlay of the manually annotated blood (ground truth), Right: green overlay of the RFC output (prediction) (Color figure online)

The best-performing Random Forest classifier was developed with 79 trees, Entrophy split criterion, maximum depth of 6, and a maximum samples ratio of 1.0. The performance evaluation of the blood pixel detection by the RFC on the test images is summarized in Table 2. The model detected the blood pixels in approximately ten milliseconds per laparoscopic image. The blood pixel detection in the test laparoscopic images with the highest and lowest Dice score are visualized in Figs. 5 and 6, respectively.

**Table 2:** Performance of blood pixel detection by RFC in means and standard deviation

Accuracy (%)	Precision (%)	Sensitivity (%)	Specificity (%)	Dice score	Area (%)
94.0 ± 2.34	39.0 ± 6.1	64.6 ± 11.3	95.7 ± 2.1	0.472 ± 0.054	173 ± 52.0

### Automated detection of a device-induced bleeding

The automated detection of device-induced bleeding was developed based on 2623 video fragments. Fifty-five video fragments were excluded as they followed a previous video fragment too closely by a rapid succession of electrosurgical device activations. Twenty-one of the included video fragments were manually classified to contain device-induced bleeding. Based on the forward selection, *Blood*_*mean*_, *Ratio*_*mean*_, and *Increase*_*max*_ were selected for bleeding detection by the LR.

**Fig. 7:**
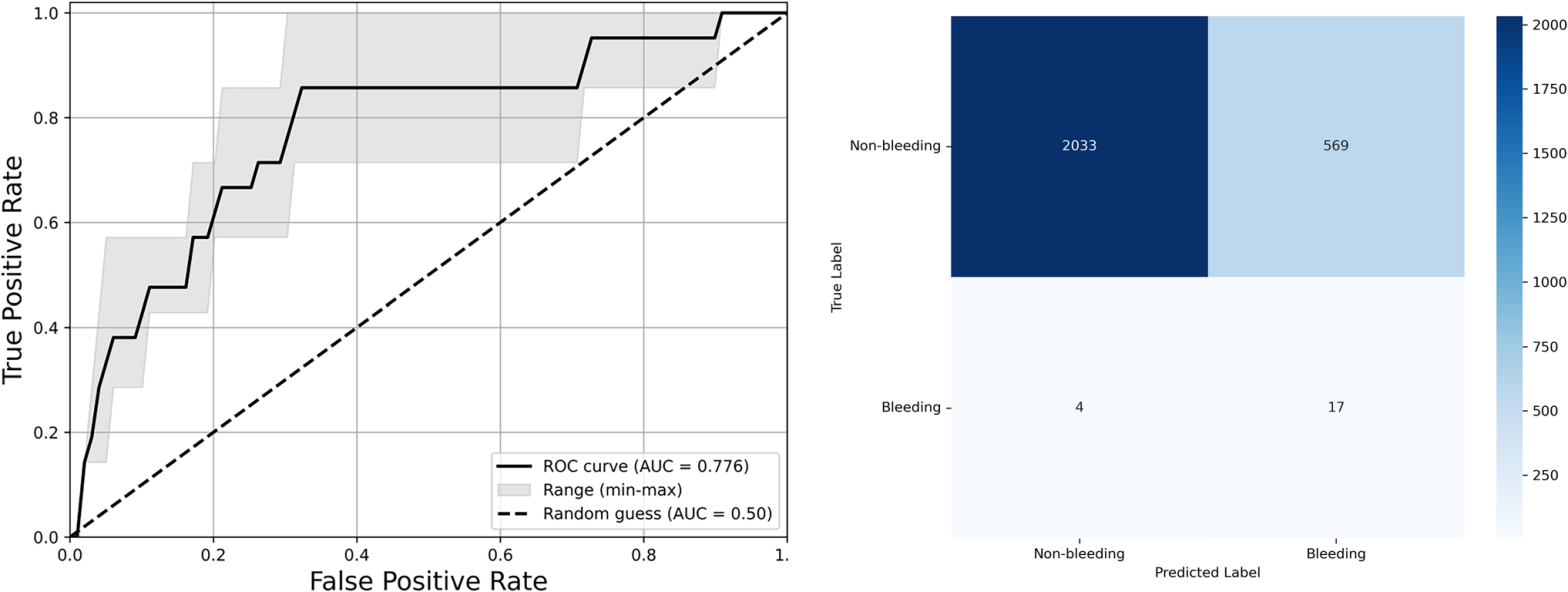
Left: the ROC curve of the best-performing LR model with mean and min–max range. Right: the confusion matrix of the logistic regression model combined over the three cross-validation folds

The final YOLO11 model configuration used for tool detection was a medium-sized model optimized for fitness, no class weights, and the first 10 layers of the model frozen. For the detection of the Enseal, it had a mAP50 of 0.962.The logistic regression model used the liblinear solver with L2 regularization and *C* = 1.0, with balanced class weighting. The detection performance for different ROI sizes is given in Table 3. The smallest ROI led to the detection with the highest ROC-AUC, precision, and sensitivity. A smaller region of interest increased sensitivity while lowering specificity. The ROC-AUC and confusion matrix of the LR model with the smallest ROI are shown in Fig. 7. Based on the confusion matrix, the negative predictive value is 99.8%.

**Table 3:** The results of the bounding box size configuration over the three folds expressed in means and min–max ranges

Bounding box size	Area under curve	Accuracy (%)	Precision (%)	Sensitivity (%)	Specificity (%)
Small	**0.776 [0.713–0.857]**	78.2 [68.8–95.1]	**4.6 [2.2–9.1]**	**81.0 [57.1–100]**	78.1 [68.6–95.4]
Medium	0.739 [0.707–0.777]	80.6 [73.3–85.7]	3.0 [2.5–3.2]	71.4 [57.1–85.7]	80.7 [73.2–85.9]
Big	0.680 [0.645–0.740]	82.7 [73.8–88.6]	3.0 [1.7–4.0]	57.1 [57.1–57.1]	82.9 [74.0–88.8]
None	0.724 [0.679–0.772]	**86.3 [83.8–90.4]**	3.6 [3.4–3.7]	61.9 [42.9–71.4]	**86.5 [83.9–90.8]**

Bold value indicates the highest performance

## Discussion

In this work, we presented a machine learning-based detection of device-induced bleeding in laparoscopic videos. We showed that blood pixels can be detected by an RFC based on color representation with an accuracy of 94.0% and a Dice score of 0.472. The automated classification of video fragments detected device-induced bleeding with a 78.2% accuracy and 81.0% sensitivity. However, due to the class imbalance, the precision is 4.6%, and the negative predictive value is 99.8%. The study results indicate the methodology performs well for correctly classifying video fragments as non-bleeding but struggles to label fragments as bleeding accurately. Nevertheless, this method is a step toward quantifying the effect of electrosurgery use. This quantification would enable post-operative insights into the application, teaching surgeons to operate with optimal hemostasis and minimal tissue coagulation.

Several studies have explored the automated detection of blood pixels and bleeding in medical images. In previous studies, blood was primarily detected in wireless capsule endoscopy (WCE) videos [11]. Ghosh et al. and Yuan et al. showed promising performance for detecting blood pixels in endoscopy images [12, 13]. However, these methodologies performed poorly on laparoscopic video data [14]. On the other hand, Garcia et al. also indicated that color representations can be used to detect blood pixels in laparoscopic videos. Okomato et al. introduced the use of machine learning to classify blood pixels based on color representations. Apart from blood pixel segmentation, Marullo et al. used deep learning to detect the accumulation of blood [7]. Action segmentation of bleeding onset in laparoscopic videos using a temporal convolutional network by Wei et al. showed a ROC-AUC of 0.823 [8]. While the results are compelling, previous literature mainly focuses on blood segmentation or action segmentation in laparoscopic video. Our study differs from the current literature by focusing on the classification of tool usage using machine learning.

Despite the low precision, this is the first machine learning-based methodology to classify bleeding onset after electrosurgical device use. However, the presented methodology assumes that bleeding will be optically visible through increased blood pixels. This assumption raises several challenges. First, the onset of bleeding may not always appear this way. As a result, the methodology may not detect bleeding in the case of an occluding anatomic structure or when detection is performed in a bloody operating field. Second, the location of the drawn ROI based on tool detection may shift from the initial activation site due to camera motion, influencing the quantification of change in blood pixels. This shift could be compensated for using a grid keypoint detector such as tracking any point (TAP) [15]. Third, due to this methodology setup, the performance of bleeding recognition highly depends on the reliability with which the blood pixels are recognized. However, automatic recognition of blood pixels in laparoscopic videos has proven difficult. On the other hand, improving the current blood pixel detector will directly contribute to better recognition of device-induced bleeding. An alternative method would be to classify the video fragments directly. While still leveraging the advantages of video fragment classification, this direct classification eliminates the intermediate quantification of blood pixels. This direct approach could be performed by video-based classification of the video fragments [16]. Finally, the automated extraction of video fragments in this methodology requires the simultaneous acquisition of both laparoscopic video and energy data. A constructive integration of both information sources will be required for a broader application of this methodology. Fortunately, this aligns with current developments of the future digital operating room, where all equipment will be integrated [17].

In addition to machine learning-based bleeding recognition, this study presented a novel way to analyze laparoscopic videos. The simultaneous recording of energy and video data enabled the automated extraction of frames around electrosurgical use, which allowed the distinction of specific sections in the laparoscopic video. This extraction brought two advantages. First, it facilitated the annotation of whether bleeding occurred after tool activation. Instead of watching the full laparoscopic video, the annotation involved reviewing short video fragments of tool activation. Second, extracting the frames greatly helped the detection algorithm as it was already focused on the relevant moment. The temporal assistance ensured the algorithm only had to perform a classification. In future studies, the automated recognition of electrosurgery moments can be used for other downstream tasks (e.g., tool movements during activation). In addition, the methodology can be applied on a larger scale as it is independent of the procedure and diathermy type. Therefore, the dataset can easily be expanded, or an analysis can be performed on the bleeding detection performance in other procedures. It is important to note that the presented bleeding detection methodology only focuses on recognizing bleeding due to electrosurgical device activations. However, surgical instruments are used dynamically, where the tip is used for blunt dissection. Therefore, a more complete view of hemostasis during surgery would also include recognizing bleeding resulting from these tissue manipulations. To recognize bleeding due to this alternative instrument use, detection will have to focus on the entire laparoscopic video. A convolutional neural network can perform this recognition by performing temporal action segmentation [8].

The performance of the blood pixel detection seemed to be influenced by certain impeding factors and the difficulty of reaching a consensus on pixel classification. As shown in Figs. 5 and 6, these factors include vessels, differences in camera lighting creating light and dark regions, and different levels of mixtures between blood and bodily fluids. Furthermore, the color of a tissue depends on its position relative to the camera and surrounding tissue. These factors affect the model as it was developed based on pixel information about blood and not the representation of anatomy (i.e., spatial information). As a result, the methodology does have the advantage that it is less dependent on the domain (i.e., frames of diaphragmatic hernia repair) on which it was developed. This approach facilitates the application of blood pixel recognition and, thus, bleeding detection within other procedures. With more annotated frames, a deep learning approach is likely more applicable and could be adopted to increase blood recognition. Despite the mentioned limitations, the blood pixel detector could also be used for other purposes. For instance, it could be used to automatically classify the overall bloodiness of the video. The accumulation of blood in a laparoscopic video provides insight into the overall course of the procedure and would be an addition to the current surgical skills assessment [5].

The limited precision and occasional false negatives of the bleeding detection model can be attributed to challenges in blood pixel detection. Inconsistent recognition of blood pixels, caused by complex anatomy, close-up views, or challenging lighting conditions, contributed to both types of errors. In non-bleeding fragments, this led to false positives due to overestimation of blood presence. In the four missed bleeding events, the model failed to detect bleeding because the actual change in blood pixels was minimal. In these subtle cases, even small misclassifications led to incorrect predictions. These inconsistencies likely resulted from reduced visibility around the tool tip or slight displacement of the region of interest due to camera motion. Since the model determines bleeding based on the difference in detected blood pixels before and after activation, such variability has a larger impact in minor bleedings. In more pronounced cases, the model is more robust to small errors in pixel classification. Threshold tuning might slightly reduce false positives, but the substantial overlap in feature values between minor bleeding and non-bleeding fragments limits its overall effectiveness. Clinically, high sensitivity is crucial for avoiding missed bleeding events, even if it increases the number of false positives. Future improvements should focus on enhancing blood pixel detection to improve both sensitivity and precision.

Future studies should focus on developing device-induced bleeding detection on a larger dataset. In our study, automated bleeding detection was developed and validated on a single procedure type. In addition, the number of device-induced bleeding limited this study. The onset of bleeding due to diathermic tool activations is very rare; in our case, less than 1% of the tool activations. The rare occurrence in our study resulted in a general lack of positive samples, leading to a skewed dataset. The limited number of positive cases could have induced a bias. Furthermore, this imbalance caused a precision of 4.6%, despite the model’s relatively high sensitivity and specificity. The limited number of positive cases also restricted the utility of optimizing the bleeding detection model for F1-score or precision-recall curve (PR AUC), as such metrics were highly sensitive to small fluctuations in detection outcomes. In preliminary experiments, optimizing for these metrics led to noticeable declines in specificity without substantial benefit to the precision, undermining the model’s ability to correctly identify non-bleeding events. Therefore, ROC-AUC was chosen as the primary optimization criterion to balance sensitivity and specificity across the imbalanced dataset. With access to a more balanced dataset, future work could consider optimizing for the F1-score or PR AUC to better prioritize the detection of rare but clinically significant bleeding events. Laparoscopic videos of many different procedures will obtain a more diverse representation of device-induced bleeding. This data would enable the development of a more robust and comprehensive performance assessment.

## Conclusion

The presented work on device-induced bleeding detection is a step toward the quantification of the effect of electrosurgery use, which will improve the current surgical skills assessment. We showed a machine learning-based methodology that accurately identifies video fragments without bleeding but struggles to identify device-induced bleeding precisely. Future work should focus on developing bleeding detection in a larger, more diverse dataset to assess whether the current challenges stem primarily from data limitations or whether methodological refinements are also required.
